# The impact of sensorimotor experience on affective evaluation of dance

**DOI:** 10.3389/fnhum.2013.00521

**Published:** 2013-09-03

**Authors:** Louise P. Kirsch, Kim A. Drommelschmidt, Emily S. Cross

**Affiliations:** ^1^Wales Institute for Cognitive Neuroscience, School of Psychology, Bangor UniversityBangor, Gwynedd, UK; ^2^Behavioural Science Institute, Donders Institute for Brain, Cognition and Behaviour, Radboud University NijmegenNijmegen, Netherlands

**Keywords:** aesthetics, neuroaesthetics, training, motor learning, observational learning, dance

## Abstract

Past research demonstrates that we are more likely to positively evaluate a stimulus if we have had previous experience with that stimulus. This has been shown for judgment of faces, architecture, artworks and body movements. In contrast, other evidence suggests that this relationship can also work in the inverse direction, at least in the domain of watching dance. Specifically, it has been shown that in certain contexts, people derive greater pleasure from watching unfamiliar movements they would not be able to physically reproduce compared to simpler, familiar actions they could physically reproduce. It remains unknown, however, how different kinds of experience with complex actions, such as dance, might change observers' affective judgments of these movements. Our aim was to clarify the relationship between experience and affective evaluation of whole body movements. In a between-subjects design, participants received either physical dance training with a video game system, visual and auditory experience or auditory experience only. Participants' aesthetic preferences for dance stimuli were measured before and after the training sessions. Results show that participants from the physical training group not only improved their physical performance of the dance sequences, but also reported higher enjoyment and interest in the stimuli after training. This suggests that physically learning particular movements leads to greater enjoyment while observing them. These effects are not simply due to increased familiarity with audio or visual elements of the stimuli, as the other two training groups showed no increase in aesthetic ratings post-training. We suggest these results support an embodied simulation account of aesthetics, and discuss how the present findings contribute to a better understanding of the shaping of preferences by sensorimotor experience.

## Introduction

Human interest in aesthetics has been present for millennia, with some of the earliest evidence coming from the Palaeolithic cave paintings in Lascaux and the so-called Venus figurines (De Smedt and De Cruz, 2013). Until recently, the study of aesthetics resided within the humanities, as philosophers, ethnographers and artists grappled with questions concerning what it meant for an object, song, poem, or dance to be considered beautiful or aesthetically pleasing. Only recently has academic interest in aesthetics broadened to also include scientific studies. In particular, neuroscientists and experimental psychologists have begun to study the cognitive and brain processes underlying a perceiver's aesthetic experience when beholding an artwork (Zeki and Lamb, 1994; Ramachandran and Hirstein, 1999; Vartanian and Goel, 2004).

A considerable number of researchers have been interested in exploring the behavioural consequences or neural substrates of aesthetic evaluation of static, visual artworks, such as paintings and sculpture (Berlyne, 1974; Cela-Conde et al., 2004; Kawabata and Zeki, 2004; Leder et al., 2004; Jacobsen et al., 2006). Far less attention has been devoted to exploring the brain and behavioral manifestations of the aesthetics of performing arts, such as theater and dance. We argue that dance is a particularly rich art form to investigate due to an equally strong reliance upon a dancer's creative and artistic sensibilities as well as his or her physical abilities. Moreover, dance is the only form of art based solely on movement of the human body. As such, behavioral and neuroscientific methods are starting to offer new insights into subjective and objective features of the relationship between movement and cognition, including action perception coupling and the perceiver's aesthetic experience of watching dance (Bläsing et al., 2012; Cross and Ticini, 2012).

Several neurocognitive investigations have incorporated dance into experimental paradigms to advance knowledge of how we perceive others' bodies in action, as well as how an observer's action experience influences perception of others' actions. Through use of neuroimaging (Calvo-Merino et al., 2005, 2006; Orgs et al., 2008), behavioral (Calvo-Merino et al., 2010; Stevens et al., 2010; Jola et al., 2012b), and combined neuroimaging and behavioral approaches (Brown et al., 2006; Cross et al., 2006, 2009a,b), these studies demonstrate how being in possession of a highly skilled movement repertoire influences perception of other people in motion (for a comprehensive overview, see Bläsing et al., 2012). One relevant strand of scientific inquiry that has used dance as a medium for understanding links between perception and action focuses on the aesthetic value of a movement to an observer (Cross and Ticini, 2012). Calvo-Merino et al. (2008) did this by investigating brain processes that underlie dance-naïve participants' aesthetic experience when watching dance. They identified what kinds of movements were most appealing to spectators and brain areas that showed greater activation when spectators watched movements that were enjoyable to observe compared to those that were less enjoyable. The authors found that visual and sensorimotor brain areas play a role in an automatic aesthetic response when viewing dance movements that are rated as enjoyable to watch.

In a subsequent fMRI study, Cross et al. (2011) aimed to draw together earlier research on action experience with questions about aesthetics by quantifying the relationship between observers' ability to physically perform dance movements and the degree to which they liked watching them. In this study, participants rated their perceived physical ability to perform dance movements (after Cross et al., 2006) and also gave an aesthetic rating of each dance movement on the like-dislike dimension of Berlyne's (1974) aesthetic evaluation scale. The behavioral data from this study showed that participants liked movements more that they perceived as difficult to perform. This result suggests that lesser embodiment (or perceived physical ability) of an observed action is associated with greater enjoyment when watching that action. While the relationship between physical familiarity and enjoyment was very clear in the Cross et al. (2011) study, this finding stands in strong contrast with a number of other experimental investigations into the relationship between familiarity and enjoyment of a stimulus. In non-dance domains, a consistent finding is that individuals tend to like objects, paintings, text, and even abstract visual stimuli more when they are familiar with them (Sluckin et al., 1982; Hekkert et al., 2003; Jacobsen et al., 2006; Bohrn et al., 2013). Such discrepant findings from the dance and non-dance domains of experimental aesthetics research underscore the need to clarify the relationship between an observer's aesthetic experience and familiarity in the physical domain. The primary aim of the present study was to clarify this relationship.

While it has been shown that physical experience with complex, full-body dance actions modulates structure and function within the human brain (Calvo-Merino et al., 2005, 2006; Cross et al., 2006, 2009a,b; Hänggi et al., 2010), it remains unclear whether and how these changes might be correlated with changes in aesthetic preference. Put in other words, it is unknown how increasing an observer's physical experience with dance movements might change his or her aesthetic response to watching those same movements. In non-dance domains, several studies have demonstrated that acquired expertise influences aesthetic judgments. Behavioral studies have shown that the level of an observer's expertise modulates his or her aesthetic evaluation of artworks (Zajonc, 1968; Sluckin et al., 1982; Schmidt et al., 1989; Hekkert and van Wiering, 1996), and brain imaging experiments have confirmed that acquired expertise is associated with changes in brain structures underlying perceptual and memory processes (Bangert et al., 2006). Together, these studies suggest that an art-viewer's expertise changes how works are perceived and judged. To date, it remains unknown how physical experience might shape a viewer's aesthetic experience of watching dance.

Based on the evidence reviewed above, it seems likely that learning to perform a particular dance movement could influence an observer's aesthetic experience of watching that movement. Montero (2012) describes this situation in behavioral terms. She maintains that dance training can facilitate a kinesthetic experience when watching dance without which some aesthetic aspects of dance performance, such as grace, power, and precision, may go unnoticed. Thus, Montero argues, physical expertise facilitates a more differentiated view on dance performances. The present study attempts to directly address the link between physical ability and aesthetic experience using a dance-training paradigm. Our primary aim was to quantify how the relationship between these two variables is manifest behaviorally. By using a popular video game system that teaches players to mirror hip-hop/popular dance sequences performed by avatars, we controlled for specific features of the physical stimuli and participants' training experience, including movement, music, costumes, and background. Thus, our approach moves a step beyond the short isolated dance clips with minimal costume or setting information used in most prior studies that have used dance to study psychological or neuroscientific questions (c.f., Bläsing et al., 2012), and helps to create a more ecologically-valid, natural performance and spectator experience (c.f., Jola et al., 2012a,b).

Participants without prior dance experience were split into three training groups: a group that physically practiced several dance sequences (physical training group), a control group that simply watched and listened to the dance training music videos (which included dancing avatars; audiovisual experience group) and another control group that only listened to the soundtrack that accompanied the dance training music videos (auditory experience only group). The audiovisual experience group was included to examine effects of embodiment *per se* on aesthetic evaluation (as participants in this group spent the identical amount of time as the physical training group watching and listening to the video stimuli) and the auditory experience only group was included to examine the impact of increasing familiarity with music on aesthetic ratings.

Distinct predictions were formulated for how different training experience should impact aesthetic judgements in the present study. Before participants began any form of training, we expected to replicate the behavioral findings of Cross et al. (2011) by demonstrating greater liking of more difficult movements. In terms of how the training manipulation should impact perception, separate predictions were formulated for each training group. For the physical training group, two alternative predictions can be distinguished. First, if increased physical experience has the same effect as increased visual familiarity with paintings (Jacobsen et al., 2006) or conceptual familiarity with texts (Bohrn et al., 2013), then we would expect these participants to like the movements more after 4 days of dance practice. Alternatively, if the relationship between aesthetic enjoyment and physical ability reported by Cross et al. (2011) endured after several days of physical practice, then we would expect participants in the physical training group to like the movements less that they have learned to perform through physical practice. For participants in the audiovisual experience group, even without ever attempting to perform the observed sequences, we expected their ability to dance the sequences they watched throughout training to improve somewhat from this observational learning context (c.f., Mattar and Gribble, 2005; Torriero et al., 2007; Cross et al., 2009a). As a consequence of this, we expected aesthetic ratings to change in the same direction (but perhaps to a lesser degree) as those from participants in the physical training group. Finally, for participants in the auditory experience only group, we did not expect their aesthetic experience of the dance movements to change, as this type of training should not result in any changes in their ability to perform the dance movements associated with the songs listened to during training.

## Methods

### Participants

Sixty-two participants (44 females, mean age = 22.60 years; *SD* = 3.38) were recruited from Bangor University to participate in a 1-week dance training study. All participants were matched across the three different training conditions in terms of age and prior dance experience. Whereas we sought to include only participants who had no prior dance experience, it was impossible to assemble a participant sample without any reported experience with dance classes or playing dance video games. Thus, we categorized participants who had less than a half-year of formal dance training as non-dance experienced, as was done in other dance training studies (Cross et al., 2011). No differences between experienced and inexperienced participants were found in relation to the research question^1^.

**Figure 1:**
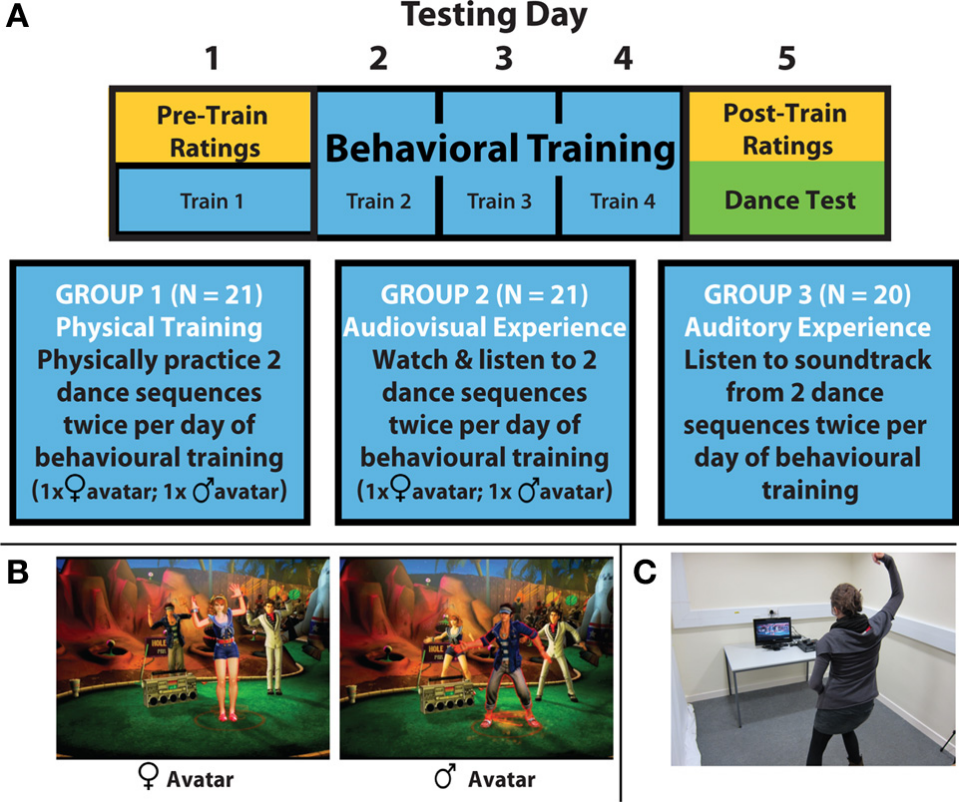
**Key elements of experimental procedures, stimuli, and apparatus. (A)** Participants partook in 5 consecutive days of testing, with pre-training ratings of the stimuli collected on the first day and post-training ratings collected on the fifth and final day of testing. Participants took part in four identical training sessions (with training experience dependent on group) on days 1–4 of the experiment. Following collection of post-training ratings on day 5, participants were asked to perform the two sequences they had some kind of experience with during training, as well as one novel sequence. **(B)** Illustrations of the female avatar and male avatar used in the Dance Central™ video game. **(C)** Laboratory set up for physical training with the Xbox Kinect™ dance video game.

Two participants were excluded from the final study sample, because they did not respond to more than 10% of the trials of the aesthetic rating tasks. Thus, the final sample size was 60 participants, with 21 participants in the physical training group, 19 participants in audiovisual experience group, and 20 participants in auditory experience only group (Figure 1A). Bangor University's ethical research committee approved all components of the study. All participants gave informed consent and received monetary compensation (in the form of gift vouchers) or course credits for their participation.

### Stimuli and apparatus

#### Physical performance measures

We chose three sequences from the database of the Xbox 360 Kinect™ game Dance Central 2 (Harmonix Music Systems, Cambridge, MA), according to the variety of dance movements and their difficulty level. The sequences lasted an average of 2:23 min (from 2:19 to 2:30 min). Dance clips were specifically chosen that contained those movements previously shown to be most appealing to dance naïve participants, such as whole body movements with significant displacement of the body in space (like jumping; Calvo-Merino et al., 2008). Furthermore, by using a classification given by the Xbox Kinect™ system (general difficulty level from 1 to 7), we chose clips with medium difficulty levels (3–5, respectively), to ensure that all participants would be able to perform them, but would still be challenged across the 4 days of training. In addition, within each general difficulty level, it was possible to specify further the level of difficulty for an individual sequence, which represents the complexity and rapidity of changing dance movements (easy, medium, and difficult). We chose a medium difficulty rating for all sequences. Two sequences were used for all three training group, and a third novel sequence was introduced on the final day of testing for all groups to attempt to physically perform a sequence with which they had no prior experience. The objective of this “surprise dance test” was to record a physical ability score for each participant, as well as to determine whether any participants who had not trained with the Kinect™ system might nevertheless have advanced dance abilities. Furthermore, this novel sequence enabled us to examine any potential carry-over effects within the physical training group (see also Cross et al., 2009a,b).

#### Affective judgment measures

For the aesthetic rating task, the two trained sequences were edited to create 20 short dance clips, 10 from each sequence, performed by both male and female avatars. Participants encountered these 20 short sequences, which ranged in length between 5 and 7 s, before and after training in a task used to assess aesthetic evaluation of the movements. Each clip was selected to contain one main movement, repeated at least twice (such as two consecutive hip swivels). As a general heuristic, all sequences of medium difficulty in the Dance Central 2 game comprise approximately 10 core movements that are repeated and arranged in different orders according to the individual song.

### Procedure

Participants arrived on the first day with no prior experience playing the Dance Central 2 video game. Participants filled in The Brunel Mood Scale (BRUMS) developed by Terry et al. (2003). This questionnaire, which is based on the Profile of Mood States, contains 24 questions divided into six respective subscales: anger, confusion, depression, fatigue, tension, and vigor. The items are answered on a 5-point Likert scale (with anchors 0 = not at all; 4 = extremely). We collected these data to ensure that any effects that might emerge across the days of training were not simply due to differences in mood unrelated to the task. We did not observe any difference of mood across groups over the week [*F*_(6.304, 185.980)_ = 0.450, *p* = 0.852]. Participants then rated the 20 short movement clips on five different questions based on an eight-point Likert scale (anchors: 1 = not at all; 8 = extremely). Movement clips (including the corresponding audio track) were presented to the participants with MATLAB R2010a Psychtoolbox3 in a random order. The five questions, based on those asked in the Cross et al. (2011) study, were presented in a random order after each sequence and designed to assess participants' affective appraisal of the movement just watched, as well as how complex and engaging they found the movement to be. The questions were “How much did you like the dance performance you just watched?,” “How complex did you find the dance performance you just watched?,” “How interesting did you find the dance performance you just watched?,” “How much would you enjoy trying to perform the movement right now?,” and “How much did you like this clip of music?”

After performing this rating task, each participant was assigned to one of the three training groups based on the assessment of his or her previous dance experience/abilities. For the physical training group, the two selected dance sequences were presented to the participants using the Xbox Kinect™ system twice in a random order (once with a female and once with a male avatar (Figure 1B); for a total of four dance sequences practiced each day). Participants stood in front of a TV screen and watched the avatar performing a dance sequence. Simultaneously, participants mirrored the movements as well as they could (Figure 1C). The Kinect™ sensor captured their movements to calculate a total score for movement accuracy, and also rated participants' overall performance on a 5-point star scale for each performed sequence. The number of stars awarded after each dance performance takes into account overall movement fluency as well as the number of well-executed movements in a row. At the end of each training day, participants from the physical training group were asked to give their subjective rating of their ability to perform the dance sequences (“How well did you know the dance sequence”) and their feelings while dancing (“How did you feel during performing the dance sequences”) on a 5-point Likert scale.

For the audiovisual experience group, the four original dance sequence videos that the physical training group trained on (two sequences each danced by a male and female avatar; including soundtracks) were presented to participants on a desktop computer with MATLAB R2010a. Participants were asked to watch the avatar perform the dance movements while sitting still. Furthermore, an attention maintenance task was added to each sequence to make sure that the participants paid attention to the stimulus material. Whenever the participants saw a fixation cross in the middle of the screen (during the video sequences), they had to push the right arrow key on the computer keyboard as fast as possible. This fixation-cross appeared randomly with a mean spacing time of 10 s.

For the auditory experience only group, participants listened twice to the soundtracks belonging to the two original video sequences. The computer screen remained black. The condition fulfilled the function of a control condition, because no visual stimuli were presented that should impact participants' dance ability. To make sure that participants paid attention to the music, random beeps was added to the music and whenever the participants heard it, they had to press the right arrow button as fast as possible. Similar to the attention task of audiovisual experience group, the beeps occurred randomly with a mean spacing time of 10 s between beeps.

On the 3 consecutive days of training (days 2–4 of the experiment), all participants came to the lab and repeated the training procedures with the same sequences they rehearsed on day 1. On the fifth and final day of testing, all participants returned for a post-training test. Each participant had to rate the same set of 20 dance clips they had rated on day 1, prior to their first training session. Then, all participants (including those who had been in the audiovisual training condition and the audio only training condition) performed three dance sequences. Each participant had to perform the two sequences they had experienced some aspects of during training, as well as a surprise, novel dance sequence to which participants had not yet been exposed in the course of the experiment. All participants performed each of the three sequences once (randomly ordered across participants). For the two groups of participants who did not train with the XBox Kinect™ system (audiovisual and auditory experience only groups), the experimenter described the task they would be performing, and care was taken so that each participant clearly understood how to reproduce the movements of the avatar and thus play the game correctly. Dance accuracy scores calculated by the Kinect™ system were recorded for the three dance sequences and all participates were asked about their perception of their ability to perform these sequences. They were asked to rate their ability to perform the dance sequences (“How well did you know the dance sequence?”) and their feelings (“How did you feel during performing the dance sequences?”) on a 5-point Likert scale for both the two trained sequences and the novel sequence.

We did not expect to find differences between groups for performance of the novel sequence, as the sequence was new to all participants (and clearly distinct from the trained sequences practiced by the physical training group). As the novel sequence was added to the post-training follow-up test after the start of the study, it was not possible to completely counterbalance all sequences beforehand. Thus, the novel sequence in the post-training follow-up test as the same for all participants^2^. At the end of the fifth testing session, participants completed a questionnaire on their attitudes and preferences about dance as well as a questionnaire about their openness to experience. As these data were collected to address another experimental question, they are not considered further in this paper.

### Data analysis

#### Physical performance

For the physical training group, participants' scores were recorded with the Kinect™ system. However, due to the complexity and somewhat opaque nature of the Kinect™ scoring system, it was necessary to devise a method for generating a numeric score that took into account all elements of performance, including raw dancing score, bonus points for number of nice moves (denoted by green stones in the final score summary), flawless moves (denoted by diamonds in the final score summary), and number of stars awarded to each performance (stars correspond to a combination of several nice or flawless moves performed consecutively)^3^. We calculated an overall score for each performed sequence based on the following algorithm: overall score = number of green stones^*^5000 + number of diamonds^*^10000 + stars^*^1000 + raw numeric score. By taking into account all the “bonus points” scored by participants, this ensured that all aspects of the performance were considered as part of the final score. Using this algorithm, we created an objective measurement of each participant in the physical training group's physical ability to perform each dance sequence at the individual training sessions. We then ran a repeated-measure ANOVA to investigate whether this group's dance scores increased across their daily training sessions.

We analyzed the subjective physical ability ratings reported each day by the participants of the physical training group, after they completed their physical practice. They were asked to rate their ability to perform the dance sequences (“How well did you know the dance sequence”) and their feelings while dancing (“How did you feel during performing the dance sequences”) on a 5-point Likert scale. The final analysis of performance ability was run on dance scores from all three groups from the post-training surprise dance test.

#### Affective judgment

In order to address our main question whether different kinds of experience with whole-body actions might change observers' affective judgments, we evaluated training effects on ratings by calculating a difference score for each participant between post-training and pre-training ratings. Standard inference statistics were used to compare the performance and judgments of the groups in critical conditions and pairwise comparisons (Bonferonni corrected) were subsequently used to look into any differences in more detail. Degrees of freedom reflect the Greenhouse-Geisser correction where sphericity has been violated. Finally, to further explore how the current data relate to behavioral findings reported by Cross et al. (2011), we computed a correlation between participants' ratings of liking and complexity both before and after training.

## Results

### Physical performance

**Figure 2:**
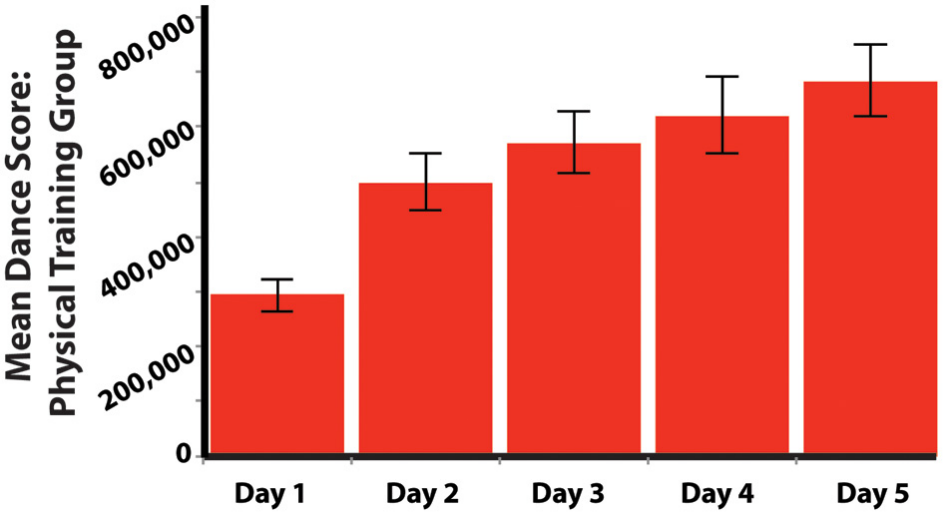
**Mean dance scores for the physical training group across the experiment.** Participants in the physical training group practiced the same two dance sequences across the 4 days of training, and then performed the same sequences one final time on the post-training dance test on Day 5. See main text for how scores were calculated based on Xbox Kinect™ output.

To determine whether participants in the physical training group improved their performance on the dance sequences they trained on for 4 consecutive days, we ran a repeated-measures ANOVA with dance score across the 4 days of training as the within-subjects variable. This analysis revealed that participants' dance scores significantly improved over the 4 days, *F*_(3, 18)_ = 11.386; *p* < 0.001, and the test of within-subjects contrasts reveals that this pattern is best captured in a linear contrast, *F*_(1, 20)_ = 21.792; *p* < 0.001 (Figure 2). Further investigation into differences between individual days of training revealed that a significant improvement was only observed from the first to the second day of training (*p* < 0.001).

**Figure 3:**
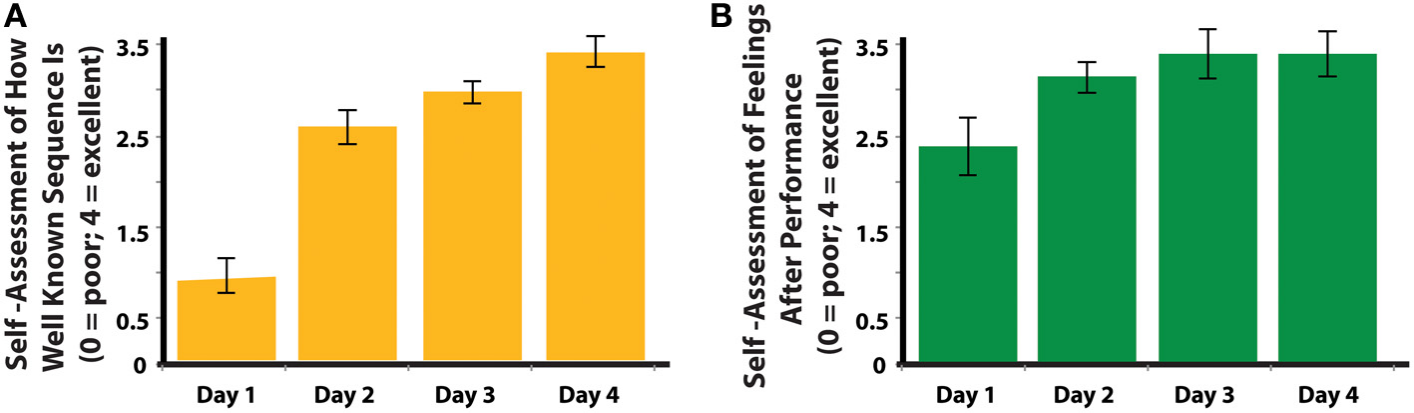
**Self-assessment by participants in the physical training group, collected each day after physical practice. (A)** Participants' assessment of how well they thought they knew the sequences they just performed. **(B)** Participants' assessment of how they felt after each day of training.

With a repeated-measures ANOVA, we analyzed participants' responses to surveys querying their subjective feelings about their performance after each training session. This analysis demonstrated a clear increase day after day of participants' subjective feelings of knowing the sequences [*F*_(4, 80)_ = 47.242; *p* < 0.001; Figure 3A]. It is of note, however, that no change was seen across training days in terms of how good they felt whilst physically performing the sequence [*F*_(1.15, 23.04)_ = 1.740; *p* = 0.201; Figure 3B]. As participants' dance scores improved (Figure 2), so did their subjective feelings of how well they know the sequences (Figure 3A).

**Figure 4:**
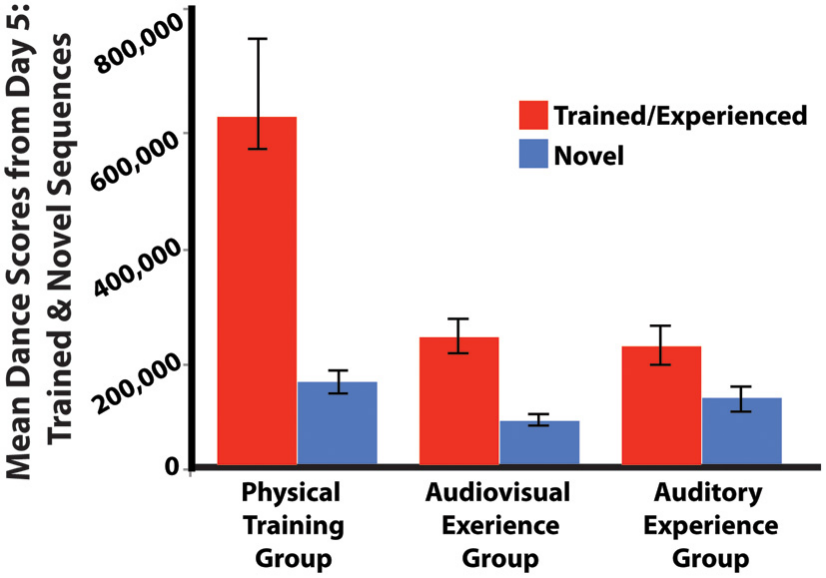
**Mean dance scores for each training group for the two dance sequences they have had some kind of prior experience with, as well as one novel dance sequence, collected on the final day of the experiment.** Only participants in the physical training group had prior experience with dancing any of the sequences.

For the third analysis of physical performance, scores were compared between all participants' performance of the two trained sequences (averaged together), and the one novel sequence, on the fifth day of the study. For the trained sequences, results show that depending on the training group, participants performed differently on the fifth day [*F*_(2, 53)_ = 28.850; *p* < 0.001; η^2^ = 0.521]. Participants from the physical training group performed better than those from the audiovisual experience and auditory experience only groups (*p* < 0.001). No differences in performance were found between the audiovisual and auditory experience only groups (*p* > 0.900). For the novel sequence, we found a weaker difference between groups, suggesting that training had less of an effect on participants' ability to perform the new sequence [*F*_(2, 53)_ = 3.565; *p* = 0.035; η^2^ = 0.119]. A significant difference emerged between the physical training and the audiovisual experience groups (*p* = 0.036) but not between the physical training group and the auditory experience only group or between the audiovisual experience group and the auditory experience only group (*p* = 0.713, *p* = 0.469, respectively; Figure 4).

As a whole, all groups showed better performance for the sequences they had some kind of experience with during the week of training (either physical, audiovisual, or auditory only) compared to the novel sequence (all *p* < 0.001). While we believe the most likely interpretation of this finding is that increased familiarity in any domain (whether motor, visual, auditory, or all three) leads to performance benefits, this finding could also be explained by some kind of inherent difference in sequence difficulty between the two trained sequences and the novel sequence. Future research could explore this possibility.

### Affective judgment

**Figure 5:**
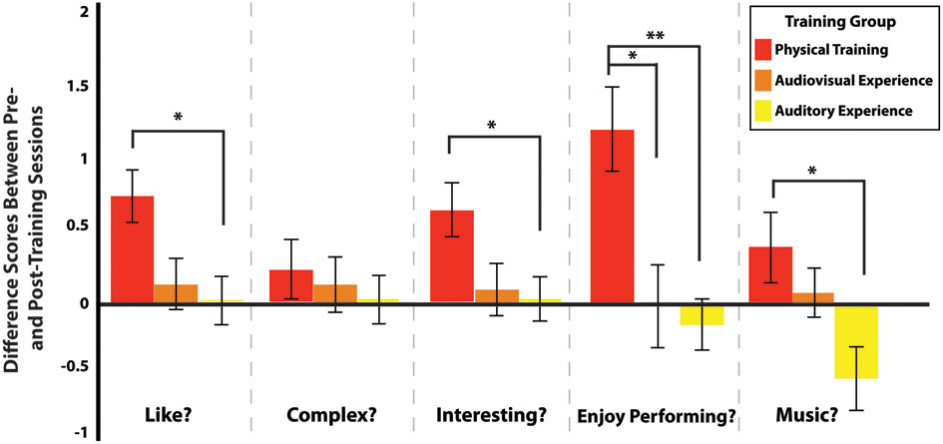
**Difference scores (calculated as post-training ratings - pre-training ratings) for each question, separated by training group.** The single asterisk (^*^) denotes significance at *p* < 0.05 and the double asterisk (^**^) denotes significance at *p* = 0.002.

The principal aim of this study was to evaluate how different kinds of experience with complex, full-body movements influence affective judgment of these movements. After computing the difference scores (post-training ratings - pre-training ratings) for each participant on short clips of the dance sequences, we conducted a multivariate ANOVA with participants' ratings as the dependent variable, the five questions as the independent variables (Like watching? How complex? How interesting? Enjoy performing? Like music?) and the three training groups as a between-subjects factor. Results indicate that training condition had a significant effect on the post-training questions of aesthetic evaluation that assessed how much participants liked the dance clip [*F*_(2, 57)_ = 3.843; *p* = 0.027; η^2^ = 0.119], how interesting they found the dance movement [*F*_(2, 57)_ = 3.830; *p* = 0.027; η^2^ = 0.118], how enjoyable participants rated performing the dance sequence [*F*_(2, 57)_ = 7.569; *p* = 0.001; η^2^ = 0.210] and how much they liked the music [*F*_(2, 57)_ = 4.269; *p* = 0.019; η^2^ = 0.130]. No effects were found on how complex participants found the dance sequences to be [*F*_(2, 57)_ = 0.337; *p* = 0.715; η^2^ = 0.012]. However, the training experience did not lead to the same effect across all questions. The biggest differences were observed between the physical performance and the auditory experience only group, with smaller or no effects seen between the physical training and audiovisual experience group (Figure 5). Compared to the auditory experience only group, participants in the physical training group show more positive responses after training to the questions about how much they like the rehearsed sequences (*p* = 0.013), how interesting they find the sequences (*p* = 0.047), and how much they like the music (*p* = 0.019). Moreover, after training, participants in the physical training group reported greater perceived enjoyment of performing the sequences compared to participants in the audiovisual experience groups (*p* = 0.018) and the auditory experience only group (*p* = 0.002).

To explore within-group effects of the training process on subjective evaluations of the dance sequences, we conducted independent MANOVAs for each training group testing for differences between pre- and post-training rating scores on the five questions. Any significant difference from zero (in the positive or negative direction) would suggest that the training procedures impacted perception. We found that participants from the physical training group significantly changed their judgments for four of the ratings [liking, *F*_(1, 19)_ = 13.816, *p* = 0.001; interest, *F*_(1, 19)_ = 11.897, *p* = 0.003; performance enjoyment, *F*_(1, 19)_ = 16.279, *p* = 0.001; and music liking *F*_(1, 19)_ = 4.419, *p* = 0.049]. In each of these instances, participants' ratings were significantly higher after training. No differences were seen between pre- and post-training ratings for participants in the audiovisual experience and the auditory experience only groups, with the exception that participants in the auditory experience only group liked the music less after training [*F*_(1, 19)_ = 4.893, *p* = 0.039].

**Figure 6:**
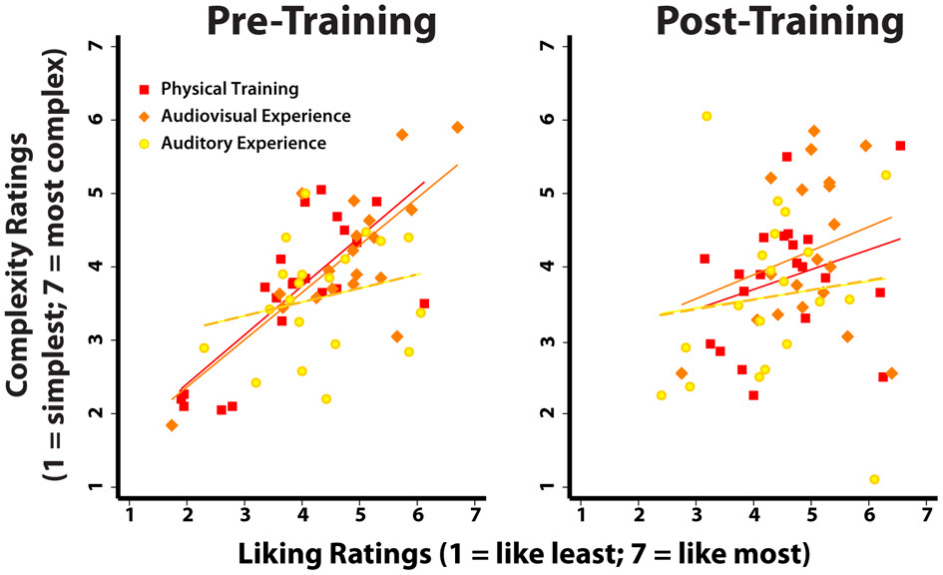
**Correlations between complexity and liking ratings, before and after training.** The left plot illustrates the positive correlation between liking and complexity before participants had any kind of experience with the dance stimuli [similar to the situation in Cross et al. (2011)]. The lines represent the regression lines of the correlation for each group, pre- and post-training (see the Results section for *r*- and *p*-values). The right plot illustrates that the positive correlation evident for the Physical Training and the Audiovisual Experience groups disappears after several days of training, such that those movements that are viewed as most complex are no longer associated with consistently high liking ratings.

The final analysis assessed whether a relationship existed between participants' ratings of liking and complexity for each movement, before and after training (Figure 6). When this analysis was run on pre-training scores, a significant correlation emerged showing that participants liked more those movements that they also rated as more complex (*r* = 0.629; *p* < 0.001). However, this positive correlation between liking and complexity disappeared in the post-training data (*r* = 0.241; *p* = 0.066). In order to better understand differences between the three training groups, we split the data into groups and ran the same correlations. The correlation between liking and complexity ratings was significant for the physical training and audiovisual experience groups (physical training group: *r* = 0.755; *p* < 0.001; audiovisual experience group: *r* = 0.708; *p* < 0.001), but not for the auditory experience only group (*r* = 0.234; *p* = 0.320). After training, the correlation was non-significant for all three groups. We ran an additional analysis to test whether the correlation coefficients differed before and after training, depending on group assignment (Raghunathan et al., 1996). We found a significant difference only for the physical training group and a marginally significant difference between pre- and post-training correlation values for the audiovisual experience group (physical training group: *z* = 2.96; *p* = 0.003; audiovisual experience group: *z* = 1.98; *p* = 0.048; auditory experience group: *z* = 0.97; *p* = 0.3297). This pattern of findings suggests that physical training most reliably impacts post-training ratings, while audiovisual experience alone has less of an influence on the relationship between liking and complexity ratings. The positive, pre-training correlation between complexity and liking is consistent with the behavioral data reported by Cross et al. (2011), and its disappearance after training will be considered in the discussion.

## Discussion

The primary aim of the present study was to investigate how different kinds of experience shape observers' aesthetic experience of watching dance. We were interested in whether increased experience with a dance sequence increases or decreases a spectator's enjoyment when watching that sequence. The hypothesis that increased familiarity is associated with increased liking (c.f., Jacobsen et al., 2006; Bohrn et al., 2013) was confirmed only for participants in the physical training group. Participants who physically practised dance sequences reported greater enjoyment when watching them, while no systematic differences in aesthetic ratings emerged among participants in the audiovisual experience or auditory experience only groups between pre-training and post-training sessions. This pattern of findings suggests that the experience of learning to embody an action may play a crucial role in how much pleasure one derives from watching that action.

On the first day of the study, we demonstrated a significant correlation between participants' ratings of liking and complexity before they had any kind of training with the movement stimuli. Specifically, we saw that participants' ratings of liking positively correlated with their ratings of perceived complexity. Considering that participants had no prior experience with the movements on the first day of the experiment, we suggest that asking them to rate the complexity of a movement is comparable to asking them how well they could reproduce a movement (although we acknowledge that these questions are not tapping identical cognitive constructs). These findings are broadly consistent with the behavioral correlation between ratings of liking and reproducibility reported by Cross et al. (2011). Cross et al. (2011) found that participants preferred watching movements they perceived as most difficult to physically reproduce. One component that might modulate the link between complexity and reproducibility is how familiar one is with a particular movement. In both Cross et al. (2011) and the current study, participants were asked to passively watch and rate dance sequences, but participants in the study by Cross et al. (2011) watched and rated the sequences during a single experimental session (whilst undergoing fMRI). In contrast, participants in the current study watched and rated the sequences on two separate occasions separated by 4 days of training. This means that all participants in the current study were more familiar with the stimuli when making the post-training ratings. Additional empirical support for this theory comes from a recent study investigating aesthetic judgments of written texts (Bohrn et al., 2013). In this experiment, participants rated how much they enjoyed reading proverbs. The authors reported that participants' ratings of familiarity and beauty were positively correlated (Bohrn et al., 2013). The current results are in line with this finding, in that the participants who became most familiar with the stimuli (the physical training group) reported the highest liking ratings after training.

### Liking what we can do: links between embodiment and aesthetics

The data from the present study suggest that there is something specific about *physical* experience, *per se*, that leads to greater enjoyment of watching that movement. We can deduce this from the fact that participants in the audiovisual and auditory experience training groups did not report increased enjoyment when watching the movements after 4 days of experience, even though they spent an identical amount of time watching and/or listening to the music videos as those in the physical training group. Previous studies with non-dancers that have explicitly studied the aesthetics of watching dance have speculated that the link between increased sensorimotor neural activity and greater enjoyment is possibly due to an implicit desire within the viewer to embody the observed movement (Calvo-Merino et al., 2008; Cross et al., 2011). Freedberg and Gallese's (2007) embodied simulation account of aesthetics posits that a perceiver's aesthetic experience of a work of art is inextricably linked to the corporeal sensations evoked by the work. In support of this theory, Freedberg and Gallese focus exclusively on static works of art in the form of paintings and sculpture, and suggest that an observer can experience “embodied resonance” when viewing a piece of art based on the content of the work itself (such as the male form struggling to emerge from the marble in Michelangelo's *Slave Called Atlas*) or via the visible traces of the artistic medium (such as the wild scattering of paint in Jackson Pollock's *Number 14: Gray*). Freedberg and Gallese (2007) thus maintain that an observer cannot help but use his or her sensorimotor system when making aesthetic evaluations of artworks. Even though Freedberg and Gallese (2007) do not consider the art form of dance in their embodied simulation account of aesthetics, the findings from Calvo-Merino et al. (2008) and Cross et al. (2011) lend concrete support to this theory by demonstrating greater engagement of sensorimotor brain regions when watching movements rated as more aesthetically pleasing. The present findings provide additional support to this theory, as they are the first to show that an observer's aesthetic experience *increases* as a result of increased embodiment.

Another factor likely to have contributed to participants in the physical training group's higher aesthetic ratings post-training is a change in their perceptual fluency of observed movements. According to Reber et al. (2004), a key determinant of aesthetic pleasure is the perceiver's processing dynamics of a stimulus. In other words, the more fluently a perceiver can process a stimulus, the more positive their aesthetic response becomes. This idea has received support from research showing that observing or performing smooth reach and grasping movements toward everyday objects results in higher aesthetic ratings of those objects compared to objects that were grasped in a more awkward way (Hayes et al., 2008). In a recent paper, Montero suggested that dance training can facilitate the perception of certain aesthetic qualities of a dance, meaning that aspects such as grace, power, and precision may go unnoticed without any physical practice (Montero, 2012). A recent study from Calvo-Merino et al. (2010) has shown that motor training affects the way movement is perceived. The authors showed pairs of point-light displays of ballet steps to both ballet experts and non-experts, and found that experts were better at identifying which displays were identical and which were different, suggesting that experts' perceptual systems are finely tuned by extensive practice. Such a finding further corroborates Montero's idea (2012) that perceptuomotor experience greatly bolsters an observers' ability to evaluate or aesthetically judge dance movement. Thus, we argue that our findings are consistent with the notion that physical practice leads to increased perceptual fluency, and this in turn positively impacts aesthetic ratings. In the present study, only those participants who became physically familiar with the movements enjoyed watching them more post-training. One possible avenue for future inquiry is whether the relationship between perceptual fluency, experience and aesthetics might relate to an overarching notion of prediction error, such that we derive more pleasure from perceiving stimuli that are predictable (or familiar). While an expanding body of research explores questions of action predictability (and how experience shapes prediction processes; c.f., Diersch et al., 2012, 2013; Cross et al., 2013), to our knowledge this issue has not yet been explored in the realm of aesthetics or affective processing. We suggest that future work might be able to draw together these themes to better elucidate the relationship between familiarity, liking and predictability.

### Limitations, implications and future directions

Several limitations of the current study warrant careful consideration. First, we acknowledge that the relationship between perceived movement complexity and liking requires further investigation and clarification. While we believe the correlation between liking and physical ability reported by Cross et al. (2011) is relevant to the correlation between liking and complexity reported in the present study, we still urge caution when considering both findings together. The present data show that complexity ratings did not change with training as much as liking ratings *increased* with training. Thus, it seems that physical familiarity engenders liking, but complexity is a more stable phenomenon that is less susceptible to change. We aim to further explore this relationship in future work.

Another aspect of the current study and other recent empirical work on dance aesthetics that could be refined is the method for evaluating the aesthetic experience of a dance observer. Although many researchers use the aesthetic rating dimensions of Berlyne (1974), we suggest that the development of more comprehensive or objective ways to measure aesthetic experience would be helpful. One possibility might be to adapt the implicit association test (c.f., Greenwald et al., 1998) as a means of sidestepping any kind of social (or artistic) desirability bias when assessing a work of art, while tapping participants' automatic appraisal of a stimulus. Ideally, any such new and improved measure of aesthetic appraisal would capture a more detailed and complete view of different perceivers' aesthetic evaluations, and could facilitate the comparison and matching of these personal, affective experiences to the underlying neural processes.

One issue to consider is whether empirical paradigms that study art-related stimuli in extremely reduced forms (such as the short dance clips used in the present study) are well suited to studying questions of aesthetics. Returning to the perceptual fluency points discussed in the previous section, it could be reasonably argued that aesthetic responses to the full dance sequences that participants perform or watch (or listen to) throughout the training week could be better interrogated through investigating responses to something more complete than 5–7 s segments of the dance movements. However, a number of studies have shown that fluency (and indeed, aesthetic) evaluations can be reliably surveyed with even very short movement stimuli in dance (e.g., Orgs et al., 2013) and non-dance (Cannon et al., 2010) domains.

However, this issue remains worthy of consideration and has not escaped the attention of other researchers whose work spans scientific and artistic domains. Jola et al. (2012b) have addressed this and related concerns through their discussion of how phenomenology and neuroscience are brought to bear on experiments involving dance as an art form. Through their work, they not only investigate observers' aesthetic responses via brain and behavioral measures during evening-length costumed dance performances in the theater (Jola et al., 2012a), but they also consider different ways in which dance and behavioral and brain science disciplines can be combined. On one hand, they point out how measures of cortical excitability during dance spectating can be used as measurement of engagement in dance (Jola et al., 2012b), but on the other hand they also argue for the additional benefit of qualitative interviews to investigate what participants actually liked and where they focused while watching dance performances. In their research, they found that people who enjoyed the dance performance gave answers that could be classified into the categories “desire to move,” feeling a “connection to the dancer” and “having an emotional response to the performance.” Their findings highlight how quantitative and qualitative research methods mutually inform one another and pave the way for developing new insights in the perception of dance.

Overall, the present study offers the next step to a better understanding of the influence of a spectator's prior experience with a movement on his or her aesthetic appraisal of that movement. We found that physical dance training led to increased ratings of enjoyment while watching dance. We have suggested several possible explanations for these results and how they inform earlier work in this field. The present findings advance the field of neuroaesthetics by giving a better understanding of the relationship between experience and the processing of stimuli (in this case, actions) by a human observer. Furthermore, by communicating this knowledge to the dance community and those involved in arts policy, these findings have the potential to aid in the development of arts outreach programs and new dance audiences. For instance, the first step in getting spectators more interested in watching dance might be to get them up and moving themselves.

### Conflict of interest statement

The authors declare that the research was conducted in the absence of any commercial or financial relationships that could be construed as a potential conflict of interest.

## Footnotes

^1^Three MANCOVAS were evaluated with experience (yes/no) as independent variables and post-measures of Question 1–5 as dependent variables. The multivariate tests revealed no significant influences of experience in any of the three groups (*p* = 0.443; *p* = 0.357; *p* = 0.101, respectively). However, these values should be interpreted with some caution due to the fact that participants' experience was coded only according to binominal variables.

^2^As the surprise dance test measure was added to the experiment after testing had already commenced, the first five participants did not perform the novel sequence in the follow-up surprise dance retest. Thus, all data reported on this measure come from a subset of 57 participants from the total sample of 60 participants.

^3^To our knowledge, definitive guide for precisely how the Xbox Kinect™ system scores movement is not available. Our attempts to contact Microsoft to get this information were unsuccessful, but ample searching of video game web forums revealed information about the relative quality of green stones and diamonds, as well as what the stars correspond to. Thus, although our scoring algorithm is not directly calculated from the automated Kinect™ numeric score generated at the end of each dance performance, it is based on accurate relative values of the video game feedback, and as all participants were scored by the same system, any bias or inaccuracies in our scoring algorithm apply equally across all participants' dance scores, and thus should not negatively impact results.
